# How a Blood Cell Intracellular Signal Keeps Blood Flowing

**DOI:** 10.1371/journal.pbio.1001385

**Published:** 2012-08-28

**Authors:** Richard Robinson

**Affiliations:** Freelance Science Writer, Sherborn, Massachusetts, United States of America

**Figure:**
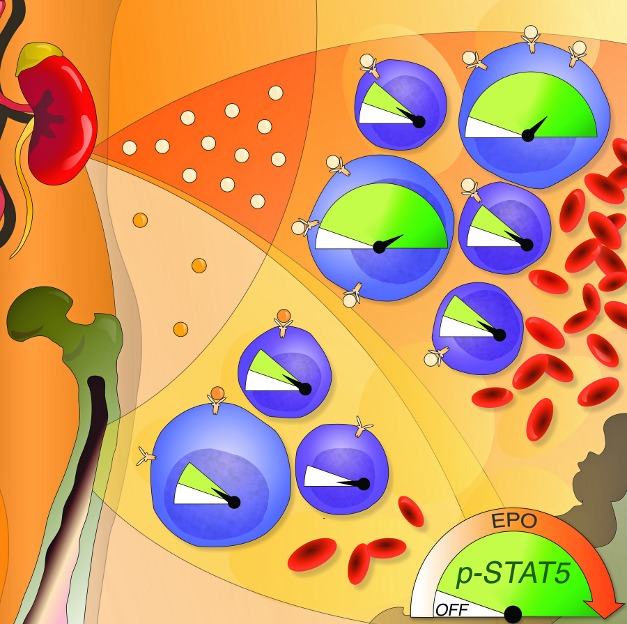
The hormone erythropoietin (Epo) stimulates differentiation of bone-marrow erythroblasts into red cells by activating a binary intracellular Stat5 signal. In hypoxic stress, higher Epo increases red cell production by generating a graded Stat5 signal that reaches high intensities in early (large) erythroblasts.

“Epo,” or erythropoietin, is not just a performance-enhancing drug for endurance athletes. It is an essential hormone that regulates the rate of erythropoiesis, or creation of new red blood cells. Basal Epo signaling ensures that such cells are made constantly at a low level, to replace worn-out cells. Certain stresses, such as hypoxia, increase Epo and lead to a steep increase in erythropoiesis, a phenomenon known to mountain climbers, who adapt to the low oxygen at high altitudes by packing more red blood cells into their blood.

The Epo signaling cascade begins when the hormone binds to the Epo receptor. This ultimately leads to phosphorylation of a transcription factor called Stat5, which turns on a suite of genes controlling cell proliferation. A key question in understanding the Epo-Stat5 system is whether and how basal and stress-induced signaling differ. In a new study in this issue of *PLOS Biology*, Emelinda Porpiglia, Merav Socolovsky, and colleagues set out to answer that question.

The authors used fluorescently labeled antibodies to tag phosphorylated Stat5 in erythropoeitic liver cells of mouse embryos. They also used a variety of cell surface markers to distinguish early versus late erythroblasts (cells that differentiate into red blood cells). They characterized three aspects of Stat5 signaling within Epo-stimulated cells: the median fluorescence from all cells within a subpopulation of erythroblasts, the fraction of those cells that were engaged in Stat5 signaling, and the level of phosphorylated Stat5 within signaling cells.

They found that Stat5 signaling was highest in precursor cells, and declined with maturation. Furthermore, precursors exhibited an “analog,” graded increase in signaling in response to rising Epo levels. This graded increase was a combination of an increase in the number of signaling cells, and an increase in the signaling intensity within each cell.

In contrast, older cells, which overall had much lower Stat5 signaling, responded to an increase in Epo by increasing the number of signaling cells, but within each cell, the signal intensity was binary, either “on” or “off,” with little change in intensity in the “on” position.

What regulates this transition from analog to binary response? In large part, they found, the ability to mount a high-intensity and graded response was due to larger amounts of Stat5 protein in earlier cells, rather than differences in Epo receptor level. When the authors added exogenous Stat5 to older cells, they too were able to signal at the higher level and in a graded way. Conversely, in cells with a mutant Epo receptor unable to bind Stat5, only basal-level, binary signaling was present, similar to the older cells.

A binary signal, the authors argue, provides an ideal way to filter out the noise at low incoming Epo signal strength, while the analog response at higher Epo concentrations provides the cell with a more dynamic ability to respond to varying levels of stress. Additionally, they found, part of the stress response was an increased production of transferrin receptor (CD71), probably to help bring in extra iron to make more hemoglobin.

Understanding this system is important not only for a better theoretical understanding of the dynamics of cell signaling. Targeting the high-level Stat5 signal, while maintaining the low-level one, may be a viable strategy for treating myeloproliferative disorders, including some types of leukemia, in which Stat5 is constitutively active.

**Porpiglia E, Hidalgo D, Koulnis M, Tzafriri AR, Socolovsky M (2012) Stat5 Signaling Specifies Basal versus Stress Erythropoietic Responses through Distinct Binary and Graded Dynamic Modalities. doi:10.1371/journal.pbio.1001383**

